# Network Traffic Classification by Program Synthesis

**DOI:** 10.1007/978-3-030-72016-2_23

**Published:** 2021-03-01

**Authors:** Lei Shi, Yahui Li, Boon Thau Loo, Rajeev Alur

**Affiliations:** 8 Eindhoven University of Technology, Eindhoven, The Netherlands; 9 Aalborg University, Aalborg East, Denmark; 10 University of Pennsylvania, Philadelphia, PA 19104 USA; 11 Tsinghua University, Beijing, China

**Keywords:** Program synthesis, Network traffic analysis, Supervised learning.

## Abstract

Writing classification rules to identify interesting network traffic is a time-consuming and error-prone task. Learning-based classification systems automatically extract such rules from positive and negative traffic examples. However, due to limitations in the representation of network traffic and the learning strategy, these systems lack both expressiveness to cover a range of applications and interpretability in fully describing the traffic’s structure at the session layer. This paper presents Sharingan system, which uses program synthesis techniques to generate network classification programs at the session layer. Sharingan accepts raw network traces as inputs and reports potential patterns of the target traffic in NetQRE, a domain specific language designed for specifying session-layer quantitative properties. We develop a range of novel optimizations that reduce the synthesis time for large and complex tasks to a matter of minutes. Our experiments show that Sharingan is able to correctly identify patterns from a diverse set of network traces and generates explainable outputs, while achieving accuracy comparable to state-of-the-art learning-based systems.

### 1 Introduction

Network monitoring systems are essential for network infrastructure management. These systems require classification of network traffic at their core. Today, network operators and equipment vendors write classification programs or patterns upfront in order to differentiate target flows such as attacks or undesired application traffic from normal ones. The process of writing these classification programs often requires deep operator insights, can be error prone, and is not easy to extend to handle new scenarios.

There have been a number of recent attempts at automated generation of classifiers for malicious traffic using machine learning[16,38,5,12] and data mining[6,28,34,39,19] techniques. These classifiers have not gained much traction in production systems, in part due to unavoidable false positive reports and the gap between the learning output and explainable operational insights[31]. The challenges call for a more expressive, interpretable and maintainable learning-based classification system.

To be specific, such challenges first come from the extra difficulties learning-based systems face in network applications compared to traditional use cases such as recommendation systems, spam mail filtering or OCR [31]. Misclassifications in network systems have tangible cost such as the need for operators to manually verify potential false reports. Due to the diverse nature and large data volumes of networks in production environments, entirely avoiding these costly mistakes by one training stage is unlikely. Therefore explainability and maintainability plays a core role in a usable learning system.

Properly representing network traffic and learnt patterns is another major difficulty. As a data point for classification purposes, a network trace is a sequence of packets of varying lengths listed in increasing timestamp order. Existing approaches frequently compress it into a regular expression or a feature vector for input. Such compression will eliminate session-layer details and intermediate states in network protocols, making it hard to learn application-layer protocols or multi-stage transactions. These representations also require laborious task-specific feature engineering to get effective learning results, which undermines the systems' advantages of automation. It can also be hard to interpret the learning results to understand the intent and structure of the traffic, due to the blackbox model of many machine-learning approaches and the lack of expressiveness in the inputs and outputs to these learning systems.

To address the above limitations, we introduce Sharingan, which uses program synthesis techniques to auto-generate network classification programs from labeled examples of network traffic traces. Sharingan aims to bridge the gap between learning systems and operator insights, by identifying properties of the traffic that can help inform the network operators on its nature, and provide a basis for automated generation of the classification rules. Sharingan does not aim to outperform state-of-the-art learning systems in accuracy, but rather match their accuracy, while generating output that is more explainable and easier to maintain.

To achieve these goals, we adopt techniques from *syntax guided program synthesis* [1] to generate a NetQRE [37] program that distinguishes the positive and negative examples. NetQRE, which stands for Network *Quantitative Regular Expressions*, enables quantitative queries for network traffic, based on flow-level regular pattern matching. Given an input network trace, a NetQRE program generates a numerical value that quantifies the matching of the trace with the described pattern. The classification is done by comparing the synthesized program's output for each example with a learnt threshold $T$. Positive examples fall above $T$. The synthesized NetQRE program serves the role of network classifier, identifying flows which match the program specifications.

Sharingan has the following key advantages over prior approaches, which either rely on keyword and regular expression generation [6,28,34,39,19] or statistical traffic analysis [16,38,5,12].

**Requires minimal feature engineering:** NetQRE [37] is an expressive language, and allows succinct description of a wide range of tasks ranging from detecting security attacks to enforcing application-layer network management policies. Sharingan can synthesize any network task on raw traffic expressible as a NetQRE program, without any additional feature engineering. This is an improvement over systems based on manually extracted feature vectors. Also, one outstanding feature of search-based program synthesis is that the only a priori knowledge it needs is information about the language itself. No task-specific heuristics are required.

**Efficient implementation:** The NetQRE program synthesized by Sharingan can be compiled, as has been shown in prior work [37], to efficient low-level implementations that can be integrated into routers and other network devices. On the other hand, traditional statistical classifiers are not directly usable or executable in network filtering systems.

**Easy to decipher and edit:** Finally, Sharingan generates NetQRE programs that can be read and edited. Since they are generic executable programs with high expressiveness, the patterns in the program reveal the stateful protocol structure that is used for the classification, which blackbox statistical models, packet-level regular expressions and feature vectors have difficulty describing. The programs are also amenable to calibration by a network operator, for example, to mix in local policies or debug.

The key technical challenge in design and implementation of Sharingan is the computationally demanding problem of finding a NetQRE expression that is able to separate positive network traffic examples from the negative ones. This search problem is an instance of the *syntax-guided synthesis*. While this problem has received a lot of attention in recent years, no existing tools and techniques can solve the instances of interest in our context due to the unique semantics of NetQRE programs, the complexity of the expressions to be synthesized and the scale of the data set of network traffic examples used in training. To address this challenge, we devised two novel techniques for optimizing the search – *partial execution* and *merge search*, which effectively achieve orders of magnitude reduction in synthesis time. We summarize our key contributions:

**Synthesis-based classification architecture.** We propose the methodology of reducing a network traffic classification problem to a synthesis from examples instance.

**Efficient synthesis algorithm** We devise two efficient algorithms: *partial execution* and *merge search*, which efficiently explore the program space and enable learning from very large data sets. Independent of our network traffic classification use cases, these algorithms advance the state-of-the-art in program synthesis.

**Implementation and evaluation.** We have implemented Sharingan and evaluated it for a rich set of metrics using the CICIDS2017 [25,7] intrusion detection benchmark database. Sharingan is able to synthesize a large range of network classification programs in a matter of minutes with accuracy comparable to state-of-the-art systems. Moreover, the generated NetQRE program is easy to interpret, tune, and can be compiled into configurations usable by existing network monitoring systems.

### 2 Overview

Sharingan's workflow is largely similar to a statistical supervised learning system, although the underlying mechanism is different. Sharingan takes labeled positive and negative network traces as input and outputs a classifier that can classify any new incoming trace. To preserve most of the information from input data and minimize the need for feature engineering, Sharingan considers three kinds of properties in a network trace: (1) all available packet-level header fields, (2) position information of each packet within the sequence, and (3) time information associated with each packet.

Specifically, Sharingan represents a network trace as a stream of feature vectors: $S = v_0, v_1, v_2, \ldots$. Each vector represents a packet. Vectors are listed in timestamp order. Contents of the vector are parsed field values of that packet. For example, we can define

$v[0] = ip.src$, $v[1] = tcp.sport$, $v[2] = ip.dst, \ldots$.

Depending on the information available, different sets of fields can be used to represent a packet. By default, we extract all header fields at the TCP/IP level. To make use of the timestamp information, we also append time interval since the previous packet in the same flow to a packet's feature vector. Feature selection is not necessary for Sharingan.

The output classifier is a NetQRE program $p$ that takes in a stream of feature vectors. Instead of giving a probability score that the data point is positive, it outputs an integer that quantifies the matching of the stream and the pattern. The program includes a learnt threshold $T$. Sharingan aims to ensure that $p$'s outputs for positive and negative traces fall on different sides of the threshold $T$. Comparing $p$'s output for a data point with $T$ generates a label. It is possible to translate $p$ and $T$ into executable rules using a compilation step.

Given the above usage model, a network operator can use Sharingan to generate a NetQRE program trained to distinguish normal and suspected abnormal traffic generated from unsupervised learning systems. The synthesized programs themselves, as we will later show, form the basis for deciphering each unknown trace. Consequently, traces whose patterns look interesting can be subjected to a detailed manual analysis by the network operator. Moreover, the generated NetQRE programs can be further refined and compiled into filtering system's rules.

### 3 Background on NetQRE

NetQRE [37] is a high-level declarative language for querying network traffic. Streams of tokenized packets are matched against regular expressions and aggregated by multiple types of quantitative aggregators. The NetQRE language is defined by the BNF grammar in Listing 1.1.

```
<classifier>::= <program> > <value>
<program> ::= <group-by>
<group-by> ::= (<group-by>)<op>|<
    feats>
            | <qre>
<qre> ::= (<qre> <qre>)<op>
          | (<qre>)*<op>
          | <unit>
<unit> ::= /<re>/
<re> ::= <re> <re>
         | (<re>)*
         | <pred>
         | _
<pred> ::= <pred> && <pred>
           | <pred> || <pred>
           | [<feat> == <value>]
           | [<feat> >= <value>]
           | [<feat> <= <value>]
           | [<feat> -> <prefix>]
<feats> ::= <feat>
            | <feats>, <feat>
<feat>  ::= 0 | 1 | 2 | ......
<op>    ::= max | min | sum
```

Listing 1.1: NetQRE Grammar

As an example, if we want to find out if any single source is sending more than 100 TCP packets, the following classifier based on a NetQRE program describes the desired classifier:

```
( ( / [ip.type = TCP] / )*sum )max|ip.src_ip > 100
```

At the top level, there are two parts of the classifier. A processing program on the left that maps a network trace to an output number, and a threshold against which this value is compared on the right. They together form the classifier. Inputs fall into different classes based on the results of the comparison.

*Group-by* expression (`<group-by>`) splits the trace into sub-flows based on the value of the specified field (source IP address in this example):

```
( ........... )max|ip.src_ip
```

Packets sharing the same value in the field will be assigned to the same sub-flow. Sub-flows are processed individually, and the outputs of which are aggregated according to the aggregation operator (`<op>`) (maximum in this example).

In each sub-flow, we want to count the number of TCP packets. This can be broken down into three operations: (1) specifying a pattern that a single packet is a TCP packet, (2) specifying that this pattern repeats arbitrary number of times, and (3) adding 1 to a counter each time this pattern is matched.

(1) is achieved by a *plain regular expression* involving *predicates*. A predicate describes properties of a packet that can match or mismatch one packet in the trace. Four types of properties frequently used in networks can be described:

1. It equals a value. For example: `[tcp.syn == 1]`
2. It is not less than a value. For example: `[ip.len >= 200]`
3. It is not greater a value. For example: `[tcp.seq <= 15]`
4. It matches a prefix. For example: `[ip.src_ip -> 192.168]`

Predicates combined by concatenation and Kleene-star form a plain regular expression, which matches a network trace considered as a string of packets.

A *unit expression* indicates that a plain regular expression should be viewed as atomic for quantitative aggregation (in this case a single TCP packet):

```
/ [ip.type = TCP] /
```

It either matches a substring of the trace and outputs the value 1, or does not match.

To achieve (2) and (3), we need a construct to both connect the regular patterns to match the entire flow and also aggregate outputs bottom up from units at the same time. We call it *quantitative regular expression* (<qre>). In this example, we use the iteration operator:

```
( / [ip.type = TCP] / )*sum
```

It matches exactly like the Kleene-star operator, and at the same time, for each repetition of the sub-pattern, the sub-expression's output is aggregated by the aggregation operator. In this case, the sum is taken, which acts as a counter for the number of TCP packets. The aggregation result for this expression will in turn be returned as an output for higher-level aggregations.

The language also supports the concatenation operator:

```
(<qre> <qre>)<op>
```

which works analogous to concatenation for regular matching. It aggregates the quantity by applying the <op> on the outputs of two sub-expressions that match the prefix and suffix.

In addition to this core language, there is a specialization for the synthesis purpose. We observe that comparing a field with values that do not appear in any of the given examples is expensive but will not produce any meaningful information. Therefore we use the relative position in the examples' value space instead of a specific value, for example, 50% instead of 3 in value space $\{1, 3, 12, 15\}$.

## 4 Synthesis Algorithm

Given a set of positive and negative examples $E_p$ and $E_n$, respectively, the goal of our synthesis algorithm is to derive a NetQRE program $p_f$ and a threshold $T$ that differentiates $E_p$ apart from $E_n$. We start with notations to be used in this section:

**Notation.** $p$ and $q$ denote individual programs, and $P$ and $Q$ denote sets of programs. $p_1 \rightarrow p_2$ denotes it is possible to mutate $p_1$ following production rules in NetQRE's grammar to get $p_2$. The relation $\rightarrow$ is transitive. We assume the starting symbol is always <program>.

$p(x)$ denotes program $p$'s output on input $x$, where $x$ is a sequence of packets and $p(x)$ is a numerical value. If $p$ is an incomplete program, i.e., if $p$ contains some non-terminals, then $p(x) = \{q(x) \,|\, p \rightarrow q\}$ is a set of numerical values, containing $x$'s output through all possible programs $p$ can mutate into. We define $p(x).max$ to be the maximum value in this set. Similarly, $p(x).min$ is the minimum value.

The synthesis goal can be formally defined as: $\forall e \in E_p, p_f(e) > T$ and $\forall e \in E_n, p_f(e) < T$.

### 4.1 Overview

Our design needs to address two key challenges. First, NetQRE's rich grammar allows a large possible program space and many possible thresholds for search. Second, the need to check each possible program against a large data set collected from network monitoring tasks poses scalability challenge to the synthesis.

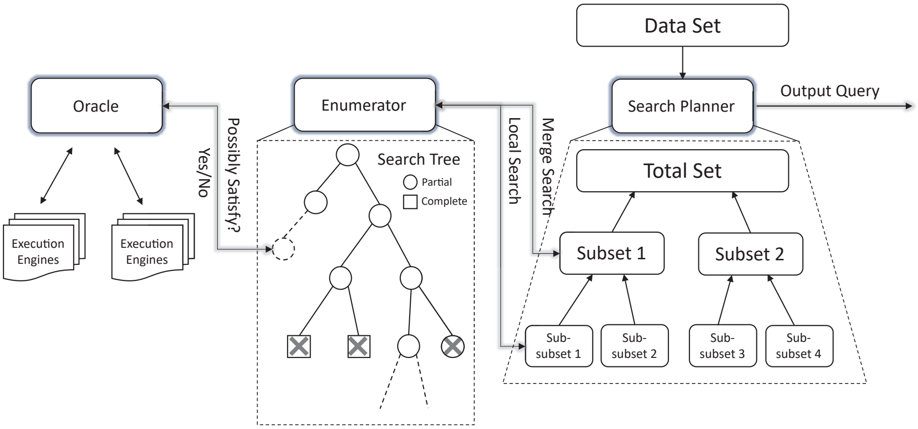

Fig. 1: Synthesizer Overview

We propose two techniques for addressing these challenges: *partial execution* (Section 4.2) and *merge search* (Section 4.3). Figure 1 shows an overview of the synthesizer.

The top-level component is the *search planner*, that assigns search tasks over subsets of the entire training data to the enumerator in a divide-and-conquer manner. Each such task is a search-based synthesis instance, where the *enumerator* enumerates all possible programs starting from $s_0$, expanded using the productions in NetQRE grammar, until one that can distinguish the assigned subset of $E_p$ and $E_n$ is found.

The *enumerator* optimizes for the first challenge by querying the distributed *oracle* about each partial program's feasibility and doing pruning early. The *oracle* evaluates partial programs using *partial execution*. The *search planner* optimizes for the second challenge by merging search results from subsets of the large training data, so as to save unnecessary checking, which we call the *merge search* strategy.

We next explain each technique in detail in the rest of this section.

### 4.2 Partial Execution

A *partial program* is an incomplete program with non-terminals. Similar to prior work making overestimation on regular expressions and imperative languages for early pruning in the search process [14,29,30], we want to evaluate a partial NetQRE program for the feasibility of all possible completions of it, so as to decide early if any of them can serve as a proper classifier for $E_p$ and $E_n$.

This process includes three main steps: (1) finding an equivalent completion $\hat{p}$ of a partial program $p$ so that evaluating $\hat{p}$ on any input $x$ is equivalent to evaluating the combination of all possible completions of $p$ on $x$, (2) efficiently evaluating $\hat{p}(x)$, (3) deciding whether to discard $p$ based on the evaluation result.

**Equivalent Completion:** Recall that we define $p(x)$ of a partial program $p$ to be the union of all $q(x)$ such that $p \to q$. Since we mainly care about outputs of positive and negative examples on different sides of a threshold, the essential information is the upper and lower bounds for $p(x)$. Therefore, the criterion for finding an equivalent completion is the bounds of $\hat{p}(x)$ should include $p(x)$ for any input $x$.

Many non-terminals have a straightforward equivalent completion. We replace (1) any uncertain numerical value with the largest or smallest possible value depending on the context, (2) any unknown predicate with *unknown*, (3) any unknown regular expression with `_*` and (4) any unknown quantitative regular expression with `(/_ _*/)*sum`. We skip the formal proof of correctness of this approach. Intuitively, the first two include all possible values at the position, and the latter two include all possible matching and aggregation strategies for a trace.

There are some non-terminals that do not have an equivalent completion, such as `<group-by>` and `<op>`. While doing enumeration, we put a complexity penalty over these non-terminals if they are not expanded, therefore encouraging earlier expansion of them so that partial execution is possible.

**Computing Ambiguity:** Notice that regular patterns naturally allow multiple matching strategies if a character(packet) in the input can match more than one predicate in the program, which is why we can estimate a set of NetQRE programs by one equivalent completion $\hat{p}$. The goal and also the major challenge in evaluating $\hat{p}(x)$ on arbitrary input $x$ is to compute the quantitative outputs from all valid matching strategies, which can grow exponentially with the input trace's length.

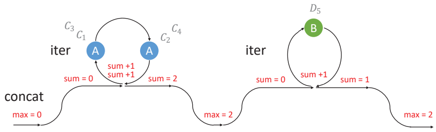

Fig. 2: Illustration of an unambiguous program. Predicate A matches packet C's while predicate B matches packet D.

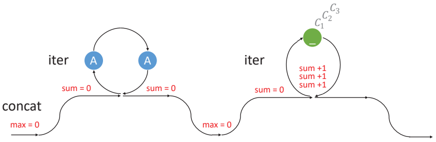

Fig. 3: Illustration of the first 3 steps of strategy one when predicate B is not yet explored.

To solve the problem of too many matching strategies, we use an approximation: merging "close" matching strategies. Two strategies are defined to be "close" if at some step of their matching process (1) they have matched the same number of packets in the trace and (2) the last predicate they have matched is exactly the same. We explore all matching strategies simultaneously and do a merging whenever two strategies can be identified to be close. Notice that each matching strategy maintains a distinct copy of aggregation states for every `<qre>` expression. States for a same expression as well as the final results are merged into one interval.

As an example, Figure 2,3,4,5 illustrates the evaluation process of a partial program during the search for the following pattern with `CCCCD` as input:

```
( ( /AA/ )*sum ( /B/ )*sum )max
```

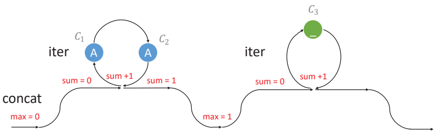

Fig. 4: Illustration of the first 3 steps of strategy two

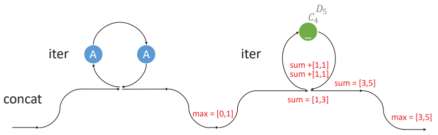

Fig. 5: Illustration of the last 2 steps of merged strategy one & two

By the properties of interval arithmetic and regular expressions, it can be proven that the approximation result strictly contains the true output range. Or more formally, $\hat{p}(x).min \leq p(x).min \leq p(x).max \leq \hat{p}(x).max$.

Intuitively, the proposed evaluation scheme works well because we only care about the boundary of outputs, which are represented by intervals as the abstract data type. We implement the execution and approximation process by the Data Transducer model proposed by [2], which consumes a small constant memory and linear time to the input trace's length given a specific program.

**Make Decision:** To make a decision regarding a partial program $p$, let $q$ be a complete program and assume there is only one pair of examples $e_p$ and $e_n$. For $q$ to accept $e_p$ and $e_n$, there must be a threshold $T$ such that $q(e_n).max < T < q(e_p).min$. Therefore, given a pair of examples $e_p$ and $e_n$, a program $q$ is correct if and only if $q(e_n).max < q(e_p).min$. When this holds, any value between $q(e_n).max$ and $q(e_p).min$ can be used as the threshold.

**Lemma 1**: There exists a correct program $q$ such that $p \rightarrow q$ only if $\hat{p}(e_n).min < \hat{p}(e_p).max$

**Lemma 2**: If $\hat{p}(e_n).max < \hat{p}(e_p).min$ then any program $q$ such that $p \rightarrow q$ is correct.

From Lemma 1, we can decide if $p$ must be rejected. From Lemma 2, we can decide if $p$ must be accepted. These criteria can be extended to more than 1 pair of examples. We will not give formal proof to the lemmas. Figures 6 and 7 show two intuitive examples for explanations of the decision making process. (but do not necessarily represent properties of real data sets). Each vertical bar represents the output range of the corresponding data point produced by the program under investigation.

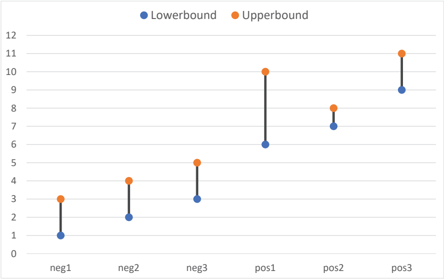

Fig. 6: A correct program found. No negative output can ever be greater than any positive output. 5.5 can be used as a threshold

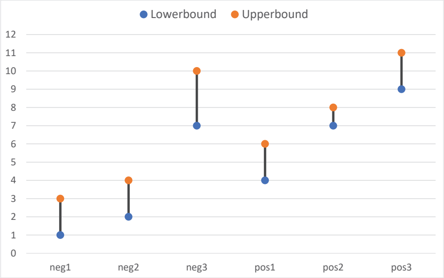

Fig. 7: A bad program. pos 1 can never be greater than neg 3.

### 4.3 Merge Search

In the rest of this subsection, we describe three heuristics for scaling up synthesis to large data sets, namely *divide and conquer*, *simulated annealing*, and *parallel processing.* We call the combination of these the *merge search* technique.

**Divide and Conquer.** Enumerating and verifying programs on large data sets is expensive. Our core strategy to improve performance is to learn patterns on small subsets and merge them into a global pattern with low overhead.

It is based on two observations: First, the pattern of the entire data set is usually shaped by a few extreme data points. Looking at these extreme data points locally is enough to figure out critical properties of the global pattern. Second, conflicts in local patterns are mostly describing different aspects of a same target rather than fundamental differences, thus can be resolved by simple merge operations such as disjunction, truncation or concatenation.

This divide and conquer strategy is captured in the following algorithm:

```
def d&c(dataset)
    if dataset.size > threshold
        subsetL,subsetR = split(dataset)
        candidateL = d&c(subsetL)
        candidateR = d&c(subsetR)
        return merge(dataset, candidateL, candidateR)
    else
        return synthesize(dataset, s0)
```

The "split" step corresponds to evenly splitting positive and negative examples. Then sub-patterns are synthesized on smaller subsets. The conquer, or "merge" step requires synthesizing the pattern again on the combined dataset. But sub-patterns are reused in two ways to speedup this search.

First, if we see a sub-pattern as an AST, then its low-level sub-trees up to certain depth threshold are added to the syntax as a new production option for the corresponding non-terminal at the sub-tree's root. They can then serve as shortcuts for likely building blocks. Second, the sub-patterns' skeletons left after removing these sub-trees are used as seeds for higher-level searches, which serve as shortcuts for likely overall structures. Both are given complexity rewards to encourage the reuse.

In practice, many search results can be directly reused from cached results generated from previous tasks on similar subsets. This optimization can further reduce the synthesis time.

**Simulated Annealing** When searching for local patterns at lower levels, we require the Enumerator to find not 1 but $t$ candidate patterns for each subset. Such searches are fast for smaller data sets and can cover a wider range of possible patterns. As the search goes to higher levels for larger data sets, we discard the least accurate local patterns and also reduce $t$. The search will focus on refining the currently optimal global pattern. This idea is based on traditional simulated annealing algorithms and helps to improve the synthesizer's performance in many cases.

**Parallelization.** Most steps in the synthesis process are inherently parallelizable. They include (1) doing synthesis on different subsets of data, (2) exploring different programs in the enumeration, (3) verifying different programs found so far, (4) executing a program on different data points during the verification.

We focus less on optimizing (1) and (2) since they are not the performance bottlenecks. We instead focus on parallelizing (3) and (4) over multiple cores. In our implementation, using 5 machines with 32 cores each, we devote one thread each to run task (1) and (2) on one machine, 64 threads on the same machine to run task (3), and 512 threads distributed over the remaining four machines to run task (4). The distributed version is approximately two orders of magnitude faster than the single-threaded version for complex tasks. Given more computing power, a proportional speedup can be expected.

## 5 Evaluation

We implemented Sharingan in 10K lines of C++ code. Our experiments are carried out in a cluster of five machines directly connected by Ethernet cables, each with 32 Intel(R) Xeon(R) E5-2450 CPUs. The frequency for each core is 2.10GHz. Arrangements of tasks are explained in the last part of Sec 4.3. We will evaluate the minimal feature engineering(5.1), accuracy(5.2), interpretability and editability(5.3), efficient implementation(5.4), and synthesis algorithm efficiency(5.5) aspects of Sharingan in order.

### 5.1 Data Preparation

We utilize eight types of attacks from the CICIDS2017 database[25,7], a public repository of benign and attack traffic used for evaluating intrusion detection systems. They cover a wide range of attack traffic including botnets, Denial of service (DoS), port scanning, and password cracking.

The data is labelled per flow by an attack type or "Benign". We learn each type of attack against benign traffic separately. To use as much data as possible, for each attack type, we use 1500 positive (attack) flows and 10000 negative (benign) flows for training, and another distinct data set of similar size for testing.

The main benefit of Sharingan in this step is the *minimal need* for feature engineering. We simply use all header fields of TCP and IP, and the inter-packet arrival time between adjacent packets in the same flow as features. In total, there are 19 features per packet and $N \times 19$ features per trace of length $N$.

In contrast, other state-of-the-art systems rely on a carefully designed feature extraction step to work well. For example, the feature vectors included in CICIDS2017 database contain 84 features extracted by the CICFlowMeter [9,13] tool for each flow, characterizing performance metrics of the entire flow such as duration, mean forward packet length, min activation time, etc. Kitsune [16] extracts bandwidth information over the past short periods as packet-level features. DECANTeR [6] uses HTTP-level properties such as constant header fields, language, amount of outgoing information, etc. as flow-level features.

### 5.2 Learning Accuracy

We next validate Sharingan's learning accuracy using the following evaluation methodology. For each individual attack type, we use the training data (attack and normal traffic) as input to Sharingan to learn a NetQRE program. The NetQRE program is then validated on the corresponding testing set for accuracy. The output of Sharingan includes a NetQRE program that maps a network trace to an integer output and a recommended range for the threshold. By modifying the threshold, true positive rate (TP) and false positive rate (FP) can be adjusted, as we will later explain in Section 5.3. We use AUC (Area under Curve) - ROC (Receiver Operating Characteristics) metric, which is a standard statistical measure of classification performance.

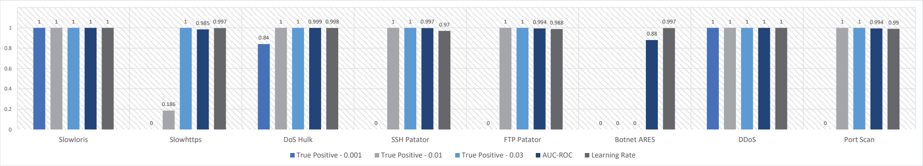

Fig. 8: Sharingan's true positive rate under low false positive rate, AUC-ROC and learning rate for 8 attacks in CICIDS2017 (higher is better)

Figure 8 contains results for eight types of attacks. Apart from AUC-ROC values, we also show the true positive rates when false positive rate is adjusted to 3 different levels: 0.001, 0.01, and 0.03. Given that noise is common in most network traffic, the last metric shown in Figure 8 is the highest achievable learning rate.

Overall, we observe that Sharingan performs well across a range of attacks with accuracy numbers on par with prior state-of-the-art systems such as Kitsune, which has an average AUC-ROC value of 0.924 on nine types of IoT-based attacks, and DECANTeR, which has an average detection rate of 97.7% and a false positive rate of 0.9% on HTTP-based malware. In six out of eight attacks, Sharingan achieves above 0.994 of AUC-ROC and 100% of true positive rate at 1% false positive rate. The major exception is Botnet ARES, which consists of a mix of malicious attack vectors. Handling such multi-vector attacks is an avenue for our future work.

### 5.3 Post-processing and Interpretation

One of the benefits of Sharingan is that it generates an actual classification program that can be further adapted and tuned by a network operator. The program itself is also close to the stateful nature of session-layer protocols and attacks, and thus is readable and provides a basis for the operator to understand the attack cause. We briefly illustrate these capabilities in this section.

**FP-TP Tradeoff** Network operators need to occasionally tune a classifier's sensitivity to false positives and true positives. Sharingan generates a NetQRE program with a threshold $T$. This threshold can be adjusted to vary the false

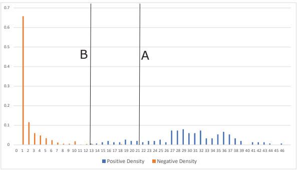

Fig. 9: Output distribution of training set(DoS Hulk)

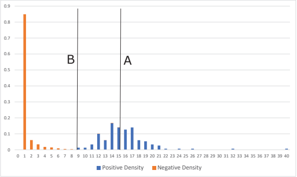

Fig. 10: Output distribution of test set(DoS Hulk)

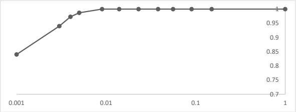

Fig. 11: ROC Curve, logarithmic scale(DoS Hulk)

positive and true positive rate. Figures 9 and 10 show the output distribution from positive and negative examples in the DoS Hulk attack. $A$ denotes the largest negative output and $B$ denotes the smallest positive output. When $A > B$, there is some unavoidable error. We can slide the threshold $T$ from $B$ to $A$ and obtain an ROC curve for the test data, as illustrated in Figure 11.

**Interpretation** We describe a learnt NetQRE program to demonstrate how a network operator can interpret the classifiers. [3] The NetQRE program synthesized by Sharingan for DDoS task above is:

```
( ( /_* A _* B _*/ )*sum /_* C _*/ )sum > 4
Where
A = [ip.src_ip->[0%,50%]]        B = [tcp.rst==1]
C = [time_since_last_pkt<=50%]
```

DDoS is a flood attack from a botnet of machines to exhaust memory resources on the victim server. The detected pattern consists of packets that start with source IP in a certain range, followed by a packet with the reset bit set to 1, and then a packet with a short time interval from its predecessor. Finally, the program considers the flow a match if the patterns show up with a total count of over 4.

The range of source IP addresses specified in the pattern possibly contains botnet IP addresses. Attack flows are often reset when the load cannot be handled or the flows' states cannot be recognized, which indicates the attack is successfully launched. Packets with short intervals further support a flood attack. Unique properties of DDoS attack are indeed captured by this program!

**Refinement by Human Knowledge** Finally, an advantage of generating a program for classification is that it enables the operator to augment the generated NetQRE program with domain knowledge before deployment. For example, in the DDoS case, if they know that the victim service is purely based on TCP, they can append `[ip.type = TCP]` to all predicates. Alternatively, if they know that the victim service is designed for 1000 requests per second, they can explicitly replace the arrival time interval with $1ms$. The modified program then is:

```
( ( /_* A _* B _*/ )*sum /_* C _*/ )sum > 4
Where
```

[3] A full list of learnt NetQRE programs can be found in our tech report https://arxiv.org/abs/2010.06135.

```
A = [ip.type = TCP]&&[ip.src_ip->[0%,50%]]
B = [ip.type = TCP]&&[tcp.rst==1]
C = [ip.type = TCP]&&[time_since_last_pkt<=1ms]
```

#### 5.4 Deployment Scenarios

We now describe three ways for network operators to deploy the output of Sharingan: (1) taking action hinted by the interpretation; (2) directly executing the NetQRE program as a monitoring system; and (3) translating the NetQRE program to rules in other monitoring systems.

Revisiting the DDoS example in Section 5.3, in the first case, the operator may refine the source IP part to find out the accurate range of attacker machines and block them.

If the NetQRE program itself is to be used as a monitoring system, its runtime system can be directly deployed on any general purpose machine. Prior work [37] has shown that NetQRE generates performance that is comparable to optimized low-level implementations. Moreover, these programs can be easily compiled into other formats acceptable to existing monitoring systems.

#### 5.5 Program Synthesis Performance

**Synthesis time:** In our final experiment, the performance of Sharingan is measured, in terms of time needed for program synthesis.

Figure 12 shows the program complexity (Y-axis) and synthesis (learning) time (in minutes). Not surprisingly, complex programs require more time to synthesize. We further observe that Sharingan is able to synthesize complex programs with at least 20-30 terms, mostly within minutes to an hour, which is practical for many real-world use cases and can be further reduced through parallelism over more machines. As a comparison, Kitsune reports training times between 8 minutes and 52 minutes on individual attacks [16], and DECANTeR reports training times between 5 hours and 10 hours on individual users' data [6].

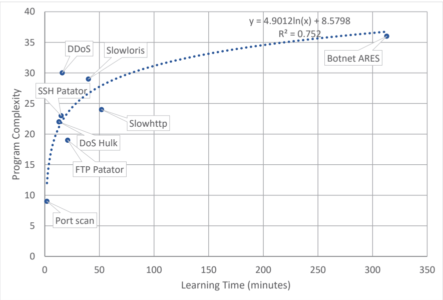

Fig. 12: Time-complexity relation

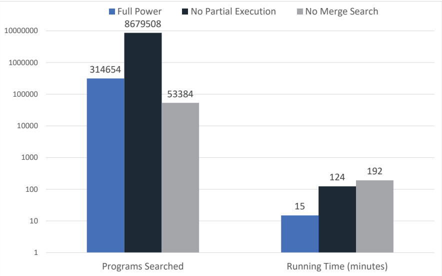

Fig. 13: Impact of optimizations on synthesis performance

**Effectiveness of Optimizations.** We explore the effectiveness of the individual optimization strategies described in Section 4. In Figure 13, we compare the synthesis time and the number of programs searched for a fully optimized Sharingan against results from disabling each optimization. SSH Patator is used as the demonstrating example since it is moderately complex.

We observe that disabling partial execution optimization makes both metrics significantly worse. Being able to prune early can indeed greatly reduce time wasted on unnecessary exploration and checking. By disabling merge search, although the number of programs searched decreases, the total synthesis time increases given the overhead of having to check each program against the entire data set. The synthesis cannot finish within reasonable time if both are disabled.

In summary, all optimization strategies are effective to speed up the synthesis process. A synthesis task that is otherwise impossible to finish within practical time can now be done in less than 15 minutes.

### 6 Related Work

**Auto-Generation of Network Configurations.** Broadly speaking, network traffic classification rule is a type of network configuration. There are other lines of research that aim at the automatic generation of different categories of network configurations. EasyACL [15] aims at synthesis of access control lists(ACL) from natural language descriptions. NetGen [24], NetComplete [10] and Genesis [32] synthesize data plane routing configurations based on SMT solvers given policy specifications. NetEgg [36] instead takes examples provided by user to generate routing configurations in an interactive way. Sharingan focuses on network traffic classification and has a different target from them.

**Other Learning-based Systems.** Apart from competing systems we explicitly compared to above, there are other learning-based systems under different settings from Sharingan.

Unsupervised learning systems are useful for recognizing outliers and other types of "abnormal" flows [17,38,35], most notably in intrusion detection systems. Its ability to differentiate unknown types of traffic from the known cannot be replaced by Sharingan. Sharingan can augment unsupervised learning systems by reducing the effort required for analyzing the reported traces.

Learning systems using state machine[18] or regular expressions for payload strings[34] as models both share the advantage of requiring minimal feature engineering. The former generates less succinct models compared to Sharingan and is typically used for verification of network protocols. The latter learns patterns at individual packet level rather than session level.

There are state-of-the-art point solutions focusing on specific scenarios rather than general-purpose network traffic classification. For example, PrivateEye focuses on detecting privacy breaches in the cloud[4]. RFDIDS solves intrusion detection challenges unique to power systems[26].

**Syntax-Guided Synthesis.** Sharingan builds on a large body of work on syntax-guided synthesis [11,21,23,20,22,29,27]. However, synthesis techniques proposed in this paper go beyond the state of the art, and have the potential to be applied to other applications of program synthesis.

Partial execution share similarity to the overestimation idea in [14] (see also follow-ups [29,30,33]), where the system learns plain regular expressions and overestimates the feasibility of a non-terminal with a Kleene-star. But no prior work proposed an overestimation algorithm for quantitative stream query languages similar to NetQRE. Nor do they consider the specification format for a classifier program with unknown numerical thresholds.

[3] proposed a divide-and-conquer strategy similar to merge search for optimizing program synthesis. It is focused on standard SyGuS tasks based on logical constraints and uses decision tree to combine sub-patterns instead of trying to merge them into one compact program. Merge search proposed in this work is not specific to Sharingan, and can be used in other synthesis tasks to allow the handling of large data sets.

Finally, there is no prior work that solely uses program synthesis to perform accurate real-world large-scale classification. The closest work concerns simple low-accuracy programs synthesized as weak learners [8], and requires a separate SVM to assemble them into a classifier.

### 7 Conclusion

This paper presents Sharingan, which develops syntax-guided synthesis techniques to automatically generate NetQRE programs for classifying session-layer network traffic. Sharingan can be used for generating network monitoring queries or signatures for intrusion detection systems from labeled traces. Our results demonstrate three key value propositions for Sharingan, namely minimal feature engineering, efficient implementation, and interpretability as well as editability. While achieving accuracy comparable to state-of-the-art statistical and signature-based learning systems, Sharingan is significantly more usable and requires synthesis time practical for real-world tasks. [4]

### Acknowledgements

We thank the anonymous reviewers for their feedback. This research was supported in part by NSF grant CCF 1763514, CNS 1513679, and Accountable Protocol Customization under the ONR TPCP program with grant number N00014-18-1-2618.
